# The successive projection algorithm as an initialization method for brain tumor segmentation using non-negative matrix factorization

**DOI:** 10.1371/journal.pone.0180268

**Published:** 2017-08-28

**Authors:** Nicolas Sauwen, Marjan Acou, Halandur N. Bharath, Diana M. Sima, Jelle Veraart, Frederik Maes, Uwe Himmelreich, Eric Achten, Sabine Van Huffel

**Affiliations:** 1 KU Leuven, Department of Electrical Engineering (ESAT), STADIUS Centre for Dynamical Systems, Signal Processing and Data Analytics, Leuven, Belgium; 2 imec, Leuven, Belgium; 3 Ghent University Hospital, Department of Radiology, Ghent, Belgium; 4 Icometrix, R&D Department, Leuven, Belgium; 5 University of Antwerp, iMinds Vision Lab, Department of Physics, Antwerp, Belgium; 6 KU Leuven, Department of Electrical Engineering (ESAT), PSI Centre for Processing Speech and Images, Leuven, Belgium; 7 KU Leuven, Department of Imaging and Pathology, Biomedical MRI/MoSAIC, Leuven, Belgium; Instituto de Investigacion Sanitaria INCLIVA, SPAIN

## Abstract

Non-negative matrix factorization (NMF) has become a widely used tool for additive parts-based analysis in a wide range of applications. As NMF is a non-convex problem, the quality of the solution will depend on the initialization of the factor matrices. In this study, the successive projection algorithm (SPA) is proposed as an initialization method for NMF. SPA builds on convex geometry and allocates endmembers based on successive orthogonal subspace projections of the input data. SPA is a fast and reproducible method, and it aligns well with the assumptions made in near-separable NMF analyses. SPA was applied to multi-parametric magnetic resonance imaging (MRI) datasets for brain tumor segmentation using different NMF algorithms. Comparison with common initialization methods shows that SPA achieves similar segmentation quality and it is competitive in terms of convergence rate. Whereas SPA was previously applied as a direct endmember extraction tool, we have shown improved segmentation results when using SPA as an initialization method, as it allows further enhancement of the sources during the NMF iterative procedure.

## Introduction

Non-negative matrix factorization (NMF) has become a widely used tool for multivariate data analysis in the fields of blind source separation and pattern recognition. NMF decomposes a non-negative input matrix *X* into the product of 2 non-negative factor matrices *W* and *H*, providing a low-rank (rank *r*) approximation:

$$\begin{array}{c} X\approx WH\;\text{with}\;X\in R_{+}^{m\times n},W\in R_{+}^{m\times r}\;\text{and}\;H\in R_{+}^{r\times n} \end{array}$$ (1)

NMF provides an additive parts-based representation of the input data. As such, it reveals the basic components which are present in the data, the so-called sources, and models each input signal (i.e. column of the input matrix) as a weighted sum of the sources. The columns of *W* represent the sources and each column of *H* contains the weights, or so-called abundances, of the sources for one particular column of *X*. The most commonly used similarity measure for *X* and its factorization is the Frobenius norm. It will also serve as the cost function for solving the optimization problem:

$$\begin{array}{c} \min_{W,H}f\left(W,H\right)=\min_{W,H}\;\frac{1}{2}{∥X-WH∥}_{F}^{2}\;,\text{such that}\;W\geq 0,\;H\geq 0 \end{array}$$ (2)

As NMF is a non-convex optimization problem, the obtained factorization will be a local rather than the global minimum of the cost function. The final result therefore depends on the initialization of the factor matrices, *W*_0_ and *H*_0_. Random initialization is the benchmark and is used in the vast majority of NMF studies. However, the quality and reproducibility of the NMF result is rarely questioned when using random initialization. As was pointed out in [1], one would have to run NMF with random initialization a sufficient number of times, then select the ‘best’ run based on some criterion (e.g. lowest Frobenius residual error), to warrant robust and reproducible NMF results. This would of course increase computation time dramatically.

Alternatively, more advanced initialization strategies have been suggested for NMF. Besides random initialization, other randomization based methods have been suggested. These methods obtain the columns of *W*_0_ by averaging a number of random columns of *X* or a subset of *X* [2]. Despite having a low computational cost and providing a more realistic first estimate of the sources compared to random initialization, these methods also suffer from a lack of reproducibility. Another group of initialization schemes is based on low-rank approximation methods of the input data matrix, such as the singular value decomposition [1, 3, 4] or independent component analysis [5]. These methods rely on the most significant low-rank components and their corresponding source vectors to initialize *W*_0_ and *H*_0_. As these methods impose constraints such as orthogonality or statistical independence to the source vectors, the non-negative structure of the input data is lost, introducing negative values into *W*_0_ and *H*_0_. Some straightforward ways of dealing with these negative values have been proposed, such as setting them to zero [1], replacing them by a mean value from the input matrix [1] or taking their absolute values [3]. Clustering algorithms have also been suggested for NMF initialization, such as spherical k-means clustering [6], fuzzy C-means clustering [7, 8] and subtractive clustering [9]. Clustering-based initialization schemes will provide more realistic source estimates compared to low-rank approximation methods, but they can become computationally expensive. Furthermore, clustering methods usually require some initialization themselves. Most of the proposed initialization methods have been compared with random initialization in terms of convergence rate and/or quality of the solution. However, different random initializations will lead to different NMF results, making it a questionnable reference. It is unclear how previous studies have coped with this lack of reproducibility.

NMF has been used in several biomedical applications, such as ECG-EMG signal unmixing [10], prostate tumor detection [11] and brain tumor segmentation [12, 13]. Brain tumor segmentation is a crucial task for planning surgical resection, for radiotherapy planning and to monitor tumor growth or shrinkage during follow-up [14]. It aims at outlining the total tumor volume as well as its main constituting tissue compartments, i.e. active tumor, necrosis and edema. NMF has been applied for tumor segmentation of multi-parametric MRI data [12, 13]. In this context, the columns of the input matrix *X* correspond to the different voxels and the rows represent the MRI parameters. The sources in *W* are interpreted as tissue-specific signatures and the abundances in *H* as the proportions of the different tissue types in each voxel. As such, NMF models each voxel’s MRI feature set as a weighted sum of the tissue-specific signatures. The abundances associated with each source can be visualised as a segmentation of the image in various tissue components and evaluated by comparison against manual expert segmentation.

The Successive Projection Algorithm (SPA) is a forward selection method which minimizes collinearity of the selected variables in vector space. It was introduced by Araújo *et al.* [15] and has been commonly used for feature selection. Several studies have considered SPA as an endmember extraction tool for hyperspectral unmixing [16, 17]. SPA is fast and reproducible, and it takes advantage of the geometrical convexity that is seen in a wide range of NMF problems [16]. To the authors’ knowledge, this is the first study in which SPA is being proposed as an initialization method for NMF. We illustrate the use of SPA initialization on 2 multi-parametric MRI datasets, applying NMF for brain tumor segmentation. The performance of SPA in terms of segmentation quality and convergence rate is compared with other common initialization methods, i.e. random initialization, non-negative double singular value decomposition (NNDSVD) and fuzzy c-means clustering (FCM). Non-deterministic methods such as random initialization are run repetitively, making sure the reported results are reproducible. Several NMF algorithms are discussed and their sensitivity to the initialization methods is investigated. The paper is further organized as follows: Section 2 describes the multi-parametric MRI datasets. Section 3 introduces the NMF methods, the SPA algorithm and the other initialization methods. NMF segmentation results comparing the different initialization methods are given in Section 4, and an in-depth discussion of the results follows in Section 5.

## Materials and methods

### Multi-parametric MRI datasets

The NMF methods are applied for brain tumor segmentation on 2 multi-parametric MRI datasets, acquired at the Ghent University Hospital (UZ Ghent) and the University Hospital of Leuven (UZ Leuven). Both datasets consisted of structural MRI, perfusion-weighted MRI, diffusion-weighted MRI and MR spectroscopic imaging, but they were acquired using a different scanning protocol. The MRI modalities provide us with complementary information about the structural, haemodynamic and biochemical properties of the brain and tumor tissue. It was shown previously that combining these MRI modalities leads to improved brain tumor segmentation [12].

The UZ Ghent dataset consisted of 21 patients who were diagnosed with a high-grade glioma. The UZ Ghent local ethics committee allowed a retrospective analysis of the data. The MR examinations were performed on a 3T Siemens Trio Tim scanner (Erlangen, Germany), using a standard 12-channel phased array head coil. A detailed description of the UZ Ghent acquisition protocol and image processing methods can be found in Appendix 1. All MRI modalities were rigidly coregistered using SPM8 (Wellcome Department of Imaging Neuroscience, University College London, UK). Skull-stripping was applied to all modalities prior to coregistration. The normalized mutual information criterion was used for coregistration [18]. All images were brought to the same spatial resolution of 1 × 1 × 3mm^3^, with cubic spline interpolation for reslicing. Only voxels within the MRSI volume of interest were included in the NMF analysis, as only these voxels had a complete set of MRI features. A total of 24 MRI features was finally obtained, making up the rows of the input matrix *X*.

The UZ Leuven patient cohort consisted of 14 high-grade glioma patients. Written informed consent was obtained from every patient before participation. MRI acquisition was performed on a 3T Philips Achieva scanner (Best, The Netherlands), using a body coil for transmission and an 8-channel head coil for signal reception. A detailed description of the UZ Leuven acquisition protocol and image processing methods can be found in the Appendix 2. A total of 29 MRI features was obtained from the multi-parametric scanning sequence. NMF analyses were restricted to one axial image slice, as the MR spectroscopic data were only acquired in 2D. Rigid coregistration with reslicing was applied as for the UZ Ghent dataset.

### NMF and initialization methods

#### NMF methods

We consider 3 different NMF problem formulations, published before in the literature, namely: single-level NMF [19], Convex NMF [20] and Hierarchical NMF (hNMF) [12]. Single-level NMF, called shortly NMF in the sequel, formulated as in Eq (2), solves for *W* and *H* in one step. Convex NMF imposes additional constraints on the source vectors in *W* and hence considers a more restricted single-level NMF problem. Hierarchical NMF, denoted as hNMF in the sequel, considers a multi-level NMF approach by splitting the initial problem hierarchically over several levels as a sequence of NMF problems in order to solve for *W* and *H*. Each NMF problem can be solved using a variety of optimisation methods. In this paper we consider 3 different algorithms for solving the single-level NMF problem: accelerated hierarchical alternating least squares NMF (aHALS NMF), gradient descent NMF (GD NMF) and projected gradient NMF (PG NMF). Convex NMF is solved using multiplicative update rules. For hNMF, we consider a 2-level approach, using aHALS NMF at each level. The proposed NMF methodologies are discussed in more detail below.

#### aHALS NMF

aHALS NMF [21] is a member of the family of alternating least squares (ALS) NMF methods. These methods rely on the observation that finding the optimal factor matrix *H* when *W* is fixed, and finding the optimal *W* when *H* is fixed, are convex problems, as opposed to the original non-convex NMF problem as defined in Eq (2). In the current study we chose to use aHALS because of its computational efficiency and fast convergence compared to other ALS methods [21].

#### GD NMF

Gradient-descent NMF (GD NMF) methods are based on non-linear least squares optimization. As these methods aim at directly minimizing the NMF cost function, they show faster convergence compared to standard NMF algorithms [22]. A transformation of variables is used to convert the constrained optimization problem in Eq (2) into an unconstrained problem, by squaring the entries of the factor matrices. The Gauss-Newton algorithm with dogleg trust region is used to solve the resulting non-linear least-squares problem [23]. The Gauss-Newton algorithm linearizes Eq (2) using Taylor’s expansion. The conjugate gradients method, which is particularly suitable for large-scale problems, can be used to calculate the gradient step from the resulting linear least squares problem at each iteration.

#### PG NMF

Lin *et al.* proposed an efficient implementation for solving the NMF problem by alternating non-negative least squares using projected gradients (PG NMF) [24]. *W* and *H* are alternatingly updated by taking steps along the negative gradient direction. Negative elements occurring throughout the iterative procedure are brought back to the non-negative orthant by setting them to zero. The “Armijo rule along the projection arc” strategy is used to determine the gradient step size.

#### Convex NMF

Convex NMF [20] imposes the constraint that the source vectors (i.e. the columns of *W*) must lie within the column space of *X*. As such, each source is a weighted sum of the data points. An auxiliary matrix *A* is introduced to define these weights:

$$\begin{array}{c} W=XA\;\text{such that}\;X\approx XAH \end{array}$$ (3)

Multiplicative update rules are defined for *A* and *H*. Without additional constraints, Convex NMF results in a sparse *H* matrix.

#### hNMF

Hierarchical NMF (hNMF) has been introduced in previous MRI studies on brain tumor characterization [12, 25]. It consists of a 2-level approach, assigning tissue types which are most similar to the same source after a first level of rank-2 NMF. Tissue-specific sources are obtained after the second level of NMF. The sources are then recombined in a final step to calculate the tissue abundances using non-negative least squares. hNMF has shown improved differentiation and segmentation of the pathologic tissue types in brain tumors compared to single-level aHALS NMF. As hNMF consists of 2 levels of NMF, the initialization methods are applied at both NMF stages.

### Initialization methods

#### Successive projection algorithm (SPA)

To better understand why SPA is suitable for finding the NMF sources, it is worthwhile to consider the NMF problem from a geometrical perspective. Let’s consider a non-negative input matrix *X* of rank 2, containing *n* data points in three-dimensional space (i.e. $X\in R_{+}^{3\times n}$). The fact that *X* has rank 2 implies that all its data points belong to a two-dimensional subspace, and they are all located in the non-negative orthant. To solve the NMF problem, we need to find 2 sources which will be located in the same two-dimensional subspace as the data points. Let’s further assume that for each source, there is at least one data point in *X* that purely contains that source (i.e. the pure-pixel assumption). In that case, it can be seen that all data points are confined within a convex cone, whose edges intersect with the pure data points. Any data point within this cone can be obtained as a weighted sum of the extreme vectors of the cone. From this geometrical point of view, NMF comes down to finding the vertices of a convex cone spanning all the data points.

For now, we assume that the pure-pixel assumption holds, meaning that some data points in *X* contain purely one source. We further assume that the sum of the abundances is not greater than 1 in any data point, which is called the sum-to-one constraint. For such input data, the NMF problem is said to be *near-separable* [26]. Under the assumptions of near-separable NMF, the data points of *X* will span a convex hull in *m*-dimensional space, and the sources correspond to the vertices of this hull. SPA fits well with this geometrical interpretation of NMF, as it aims at finding the vertices [16]. SPA works as follows: in a first step, the data point *p*_1_ with the highest *l*_2_-norm is selected, as it will correspond to a vertex of the convex hull. Next, all data points are projected onto the orthogonal complement of *p*_1_. The data point *p*_2_ with the highest *l*_2_-norm in this projected subspace will be another vertex. The next vertex *p*_3_ is found as the data point with the highest *l*_2_-norm after projection onto the orthogonal complement of *p*_1_ and *p*_2_, and so on. The columns of *W*_0_ will be formed by *p*_1_, *p*_2_, …, and *p*_*r*_. Algorithm 1 describes the SPA procedure.

**Algorithm 1: SPA**

**Input:** $X\in R_{+}^{m\times n}$, rank *r*

**Output:** Set of vectors {*p*_1_, *p*_2_, …, *p*_*r*_} ∈ *X*

 1. Initialize matrix *S* = *X*

 2. **for** *j* = *1*: *r*

 3. find index i* such that $i*=\text{ arg}{\text{max}}_{i=1,\ldots ,n}∥S(:,\;i){∥}_{2}^{2}$

 4. set *p*_*j*_ = *X*(:, i*), *s*_*j*_ = *S*(:, i*)

 5. update *S* ← $(I-\frac{s_{j}s_{j}^{T}}{∥s_{j}{∥}_{2}^{2}})S$

 6. **end**

SPA does not directly provide an initialization for the abundance matrix, *H*_0_. Based on *W*_0_, we used non-negative least squares fitting to obtain *H*_0_. For Convex NMF, an initialization of the auxiliary matrix *A*_0_ is required. Solving Eq (3) for *A*, we find:

$$\begin{array}{c} A\approx H^{T}{\left(HH^{T}\right)}^{-1} \end{array}$$ (4)

Since *A* must be non-negative, we obtain *A*_0_ as proposed in [20]:

$$\begin{array}{c} A_{0}=A^{+}+0.2E\left\langle A^{+}\right\rangle \end{array}$$ (5)

with *A*^+^ being the positive part of *A*, 〈*A*〉 = ∑_*m*,*n*_|*A*_*n*,*k*_|/∥*A*_*n*,*k*_∥_0_ and ∥*A*_*n*,*k*_∥_0_ is the number of nonzero elements in *A*. *E* is a matrix of ones with the same dimensions as *A*. *A*_0_ is similarly obtained from *H*_0_ for the other initialization methods.

#### Repetitive random initialization (rRandom)

Initialization obtained from SPA is compared with rRandom initialization, where the elements of *W*_0_ and *H*_0_ are set to uniformly distributed non-negative values between 0 and 1 at each run. We ran NMF 30 times with random initialization to obtain reproducible results. For rRandom, the final NMF result is selected as the random run with the lowest residual error as defined in Eq (2).

#### Non-negative double singular value decomposition (NNDSVD)

NNDSVD is based on 2 levels of SVD. First, the best rank-*r* approximation of the input data matrix *X* is computed based on truncated SVD:

$$\begin{array}{c} X\approx \sum_{i=1}^{r}\sigma_{i}C_{i}\;\text{such that}\;C_{i}=u_{i}v_{i}^{T} \end{array}$$ (6)

with *u*_*i*_ and *v*_*i*_ being the *i*^*th*^ left and right singular vectors of *X*. As we require a fully non-negative initialization, the positive component of *C*_*i*_, $C_{i}^{+}$ is withheld, being the nearest non-negative rank-2 approximation of *C*_*i*_. It was shown that the dominant singular triplet of $C_{i}^{+}$ can easily be obtained by decomposing the singular vectors *u*_*i*_ and *v*_*i*_ into their positive and negative components. *W*_0_ and *H*_0_ are then initialized based on the dominant singular triplet of each $C_{i}^{+}$. A detailed description of the NNDSVD algorithm can be found in [1]. Several studies have considered NNDSVD for NMF initialization [9, 27]. It must be noted that NNDSVD in its original form introduces a significant number of zero elements in *W*_0_ and *H*_0_. This can be problematic for some NMF algorithms, i.e. elements initialized at zero will remain zero in the final solution. This is for instance the case for the original multiplicative update NMF algorithm proposed by Lee and Seung [19], and also for the GD NMF algorithm that we are considering. To overcome this kind of convergence problem, we set all zero elements in *W*_0_ and *H*_0_ to a small positive value, i.e. to 1% of the mean value of the positive elements of *W*_0_ and *H*_0_, respectively.

#### Fuzzy c-means clustering (FCM)

FCM aims at partitioning the input data into *c* fuzzy clusters. The data points have a degree of belonging to each of the clusters centers, as in fuzzy logic, rather than belonging completely to just one cluster. The degree of membership of a data point *i* belonging to cluster *j* is defined by a weighting value *w*_*ij*_, which depends on a distance metric between the data point and the cluster centroid (e.g. Euclidean distance). FCM is an iterative algorithm, in which the cluster centroids and the weighting values are iteratively updated until convergence [28]. FCM has been applied as an initialization method for NMF in several studies [7, 8], with the cluster centroids being assigned to the columns of *W*_0_ and the membership weights per cluster serving as the rows of *H*_0_. It has to be noted that FCM in itself is non-deterministic, meaning that the final output will depend on the initialization. Therefore, we applied the same approach as with random initialization: run NMF with randomly initialized FCM 30 times and select the run with the lowest residual Frobenius norm as defined in Eq (2) as the final solution.

### Validation

Tissue segmentation masks are obtained by applying k-means clustering to the voxel-wise abundance values of *H*. As it is assumed that each source and its associated abundances correspond to one tissue type, we initialized the cluster centroids by setting one abundance value to 1 and all others to 0. The NMF segmentations are compared with manual segmentation performed by an experienced neuroradiologist using MRIcron [29]. The overlap between segmentations was quantified using the Dice-score:

$$\begin{array}{c} Dice_{\text{tissue}}=2\times \frac{A_{\text{tissue,NMF}}\cap A_{\text{tissue,radiol}}}{A_{\text{tissue,NMF}}+A_{\text{tissue,radiol}}} \end{array}$$ (7)

where *A*_tissue,NMF_ is the area segmented by NMF and *A*_tissue,radiol_ the area segmented by the radiologist for the same tissue type. The Dice-score is one of the most commonly used metrics to quantitatively evaluate segmentation performance in brain tumors [14, 30]. It normalizes the number of true positives to the average size of the area segmented by both methods. Dice-scores were calculated for 3 tissue classes, as defined in the BraTS challenge [30]: active tumor, the tumor core (i.e. active tumor and necrosis) and the whole tumor (tumor core and edema). Segmentation results were also assessed for direct SPA output, to compare performance of using SPA as an endmember extraction tool to using it as an initialization method. We will denote the NMF results obtained with the different initialization methods as *NMF*_*SPA*_, *NMF*_*NNDSVD*_, *NMF*_*FCM*_ and *NMF*_*rRandom*_, respectively.

## Results

**Fig 1:**
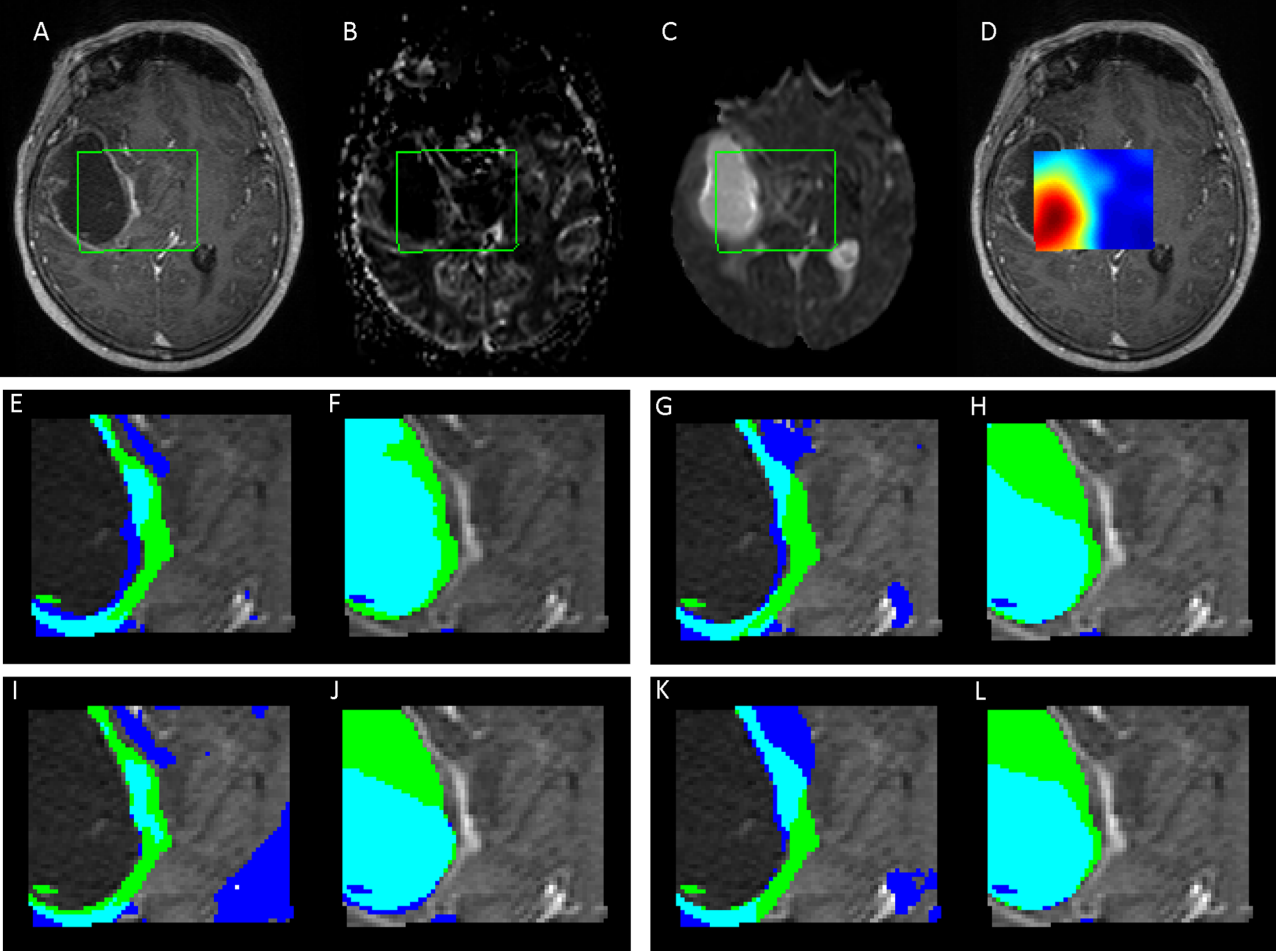
Coregistered set of MRI images. Showing T1+C(A), CBV(B), ADC(C) and Lac(D). The MRSI region of interest is marked in green. Segmentation results are shown below for active tumor and necrosis, respectively, for hNMF with each type of initialization: SPA (E,F), NNDSVD (G,H), FCM (I,J) and rRandom (K,L). NMF segmentation is shown in blue, segmentation by the radiologist in green and overlap in cyan.

Fig 1 shows an example of a coregistered image set of a glioblastoma patient. The segmentation results for active tumor and necrosis are shown for hNMF (in blue) using the different initialization methods, and are compared with the manual segmentation by the radiologist (in green). Segmentation of the active tumor region is similar for SPA, NNDSVD and rRandom, although NNDSVD and rRandom show more overlap with manual segmentation, but also some additional false positive segmentation. FCM shows a considerable oversegmentation outside of the pathologic region for active tumor. SPA obtained good segmentation correspondence for necrosis, whereas the other initialization methods show some underestimation of the necrotic area.

Table 1 shows the mean Dice-scores on the UZ Ghent dataset for SPA, NNDSVD, FCM and rRandom initialization. Looking at the results per tissue class and per NMF method, variations in mean Dice-scores among the different initialization methods are mostly below 5%. The largest difference is found for active tumor with GD NMF and Convex NMF, where *NMF*_*FCM*_ gives 5% lower Dice-scores than *NMF*_*SPA*_. The highest mean Dice-score per NMF method and per tissue class is marked in bold. SPA has the highest mean Dice-score for 12 out of 15 comparisons, NNDSVD has the highest Dice-score in only 2 cases, FCM in 3 cases and rRandom in 7 cases. Dice-scores for *NMF*_*NNDSVD*_ are lower than or at best equal to *NMF*_*SPA*_ for all tissue classes and for all NMF methods, but differences are never higher than 3%. The last column of Table 1 shows the segmentation results obtained directly from SPA output (*W*_0_, *H*_0_). The Dice-scores obtained from SPA were lower or at best equal to those from the *NMF*_*SPA*_ methods for each tissue class. Statistical significance of the higher Dice-scores for *NMF*_*SPA*_ compared to direct SPA was found in about half of the cases.

**Table 1:** Comparison of the mean Dice-scores and their standard deviation between different initialization methods for the UZ Ghent dataset. The highest Dice-score per NMF method and per tissue class is marked in bold. * indicates statistically significantly higher Dice-scores with SPA initialization compared to direct SPA endmember extraction (right column), using a one-tailed Wilcoxon signed rank test (*p* < 0.05).

		aHALS NMF	GD NMF	PG NMF	Convex NMF	hNMF	SPA (*W*_0_, *H*_0_)
**Dice active tumor [%]**	**SPA**	65 ± 13	**65 ± 21**	**66 ± 15**	**64 ± 18**	69 ± 15*	64 ± 20
	**NNDSVD**	63 ± 18	62 ± 18	64 ± 14	63 ± 19	67 ± 17	-
	**FCM**	**66 ± 14**	60 ± 21	65 ± 14	59 ± 23	69 ± 15	-
	**rRandom**	64 ± 19	63 ± 19	**66 ± 14**	63 ± 20	**70 ± 14**	-
**Dice tumor core [%]**	**SPA**	**76 ± 11**	75 ± 14*	76 ± 12*	**74 ± 14**	78 ± 12*	73 ± 13
	**NNDSVD**	74 ± 12	74 ± 11	75 ± 12	**74 ± 12**	76 ± 12	-
	**FCM**	**76 ± 11**	72 ± 14	75 ± 11	73 ± 11	77 ± 13	-
	**rRandom**	**76 ± 11**	**75 ± 11**	**76 ± 11**	73 ± 15	**78 ± 12**	-
**Dice whole tumor [%]**	**SPA**	**78 ± 12**	80 ± 11*	80 ± 12*	84 ± 10*	86 ± 8*	77 ± 13
	**NNDSVD**	**78 ± 14**	80 ± 12	78 ± 12	82 ± 10	84 ± 9	-
	**FCM**	77 ± 14	**82 ± 12**	79 ± 13	82 ± 10	84 ± 10	-
	**rRandom**	**78 ± 14**	79 ± 13	79 ± 12	82 ± 13	85 ± 8	-

Table 2 reports the mean Dice-scores on the UZ Leuven dataset for the different initialization methods. Looking at the results per NMF method and per tissue class, differences in the mean Dice-score are never higher than 5%. SPA obtains the highest mean Dice-score in 10 out of 15 comparisons. NNDSVD has the highest Dice-score in 5 cases, FCM in 7 cases and rRandom in 7 cases. Dice-scores obtained from direct SPA output were lower or at best equal to those from the *NMF*_*SPA*_ methods for each tissue class. Statistical significance of the higher Dice-scores for *NMF*_*SPA*_ compared to direct SPA could be shown in most cases, except for the active tumor region and for the whole tumor region with Convex NMF.

**Table 2:** Comparison of the mean Dice-scores and their standard deviation between different initialization methods for the UZ Leuven dataset. The highest Dice-score per NMF method and per tissue class is marked in bold. * indicates statistically significantly higher Dice-scores with SPA initialization compared to direct SPA endmember extraction (right column), using a one-tailed Wilcoxon signed rank test (*p* < 0.05).

		aHALS NMF	GD NMF	PG NMF	Convex NMF	hNMF	SPA (*W*_0_, *H*_0_)
**Dice active tumor [%]**	**SPA**	72 ± 22*	72 ± 22*	72 ± 22*	**70 ± 23**	74 ± 15*	67 ± 26
	**NNDSVD**	**72 ± 22**	68 ± 29	70 ± 21	69 ± 23	**75 ± 14**	-
	**FCM**	71 ± 21	**72 ± 22**	69 ± 22	69 ± 23	74 ± 15	-
	**rRandom**	71 ± 22	68 ± 29	71 ± 22	**70 ± 23**	74 ± 15	-
**Dice tumor core [%]**	**SPA**	84 ± 8*	83 ± 10*	85 ± 8*	83 ± 11*	85 ± 8*	79 ± 13
	**NNDSVD**	84 ± 8	80 ± 14	84 ± 8	81 ± 11	84 ± 9	-
	**FCM**	84 ± 8	**85 ± 9**	84 ± 9	**85 ± 8**	**85 ± 8**	-
	**rRandom**	**85 ± 8**	**85 ± 8**	**85 ± 7**	84 ± 7	**85 ± 7**	-
**Dice whole tumor [%]**	**SPA**	84 ± 8*	84 ± 7*	84 ± 8*	82 ± 10	86 ± 8*	80 ± 11
	**NNDSVD**	**84 ± 8**	81 ± 15	**84 ± 8**	83 ± 8	**86 ± 8**	-
	**FCM**	83 ± 8	**84 ± 7**	83 ± 8	**84 ± 8**	**86 ± 8**	-
	**rRandom**	81 ± 10	83 ± 9	81 ± 10	**84 ± 7**	**86 ± 7**	-

**Fig 2:**
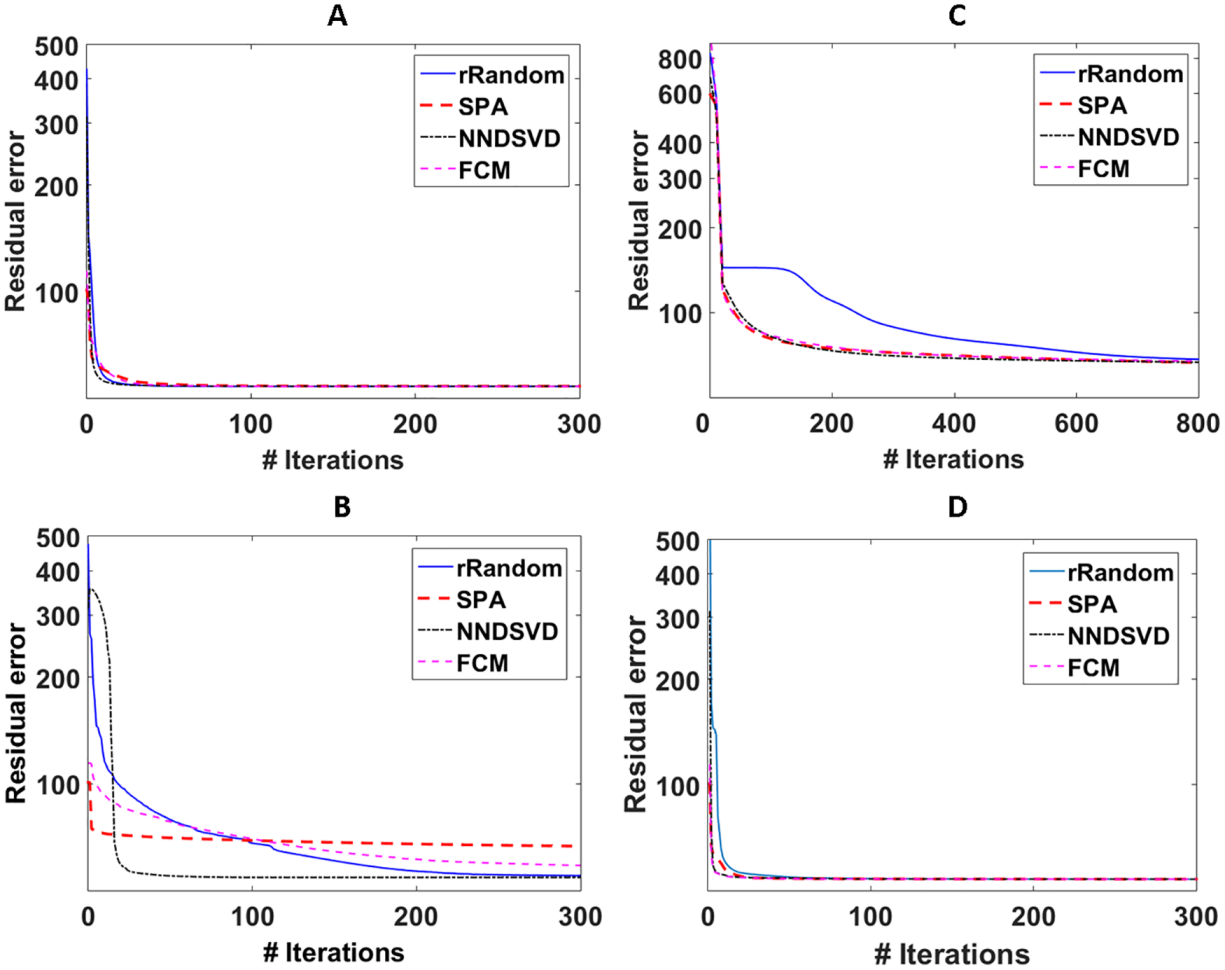
Convergence plots for aHALS NMF (A), GD NMF (B), Convex NMF (C), and PG NMF (D) with the different initialization methods. The residual error, ∥*X* − *WH*∥_*F*_, is shown on a log scale. For rRandom and FCM, the shown curve corresponds to the selected run with the lowest residual error.

Table 3 shows the mean number of iterations to convergence for the 4 single level NMF methods using the different initialization schemes. These values could not directly be reported for hNMF, as it consists of 2 levels of aHALS NMF. The same stopping criteria were applied to all NMF methods: a maximum number of iterations of 10000 and a convergence tolerance of 10^−5^ for the relative difference of two subsequent values of the residual error ∥*X*−*WH*∥_*F*_. For aHALS NMF, the mean number of iterations is below 200 for all initialization methods. Convergence rate is low for Convex NMF, where all initialization methods result in a mean number of iterations close to 9000. Only for GD NMF, we see a clear difference in convergence rate among the initialization methods. *NMF*_*SPA*_ and *NMF*_*NNDSVD*_ have the fastest convergence, with a mean number of iterations of 218 and 175, respectively. *NMF*_*rRandom*_ requires on average 418 iterations to reach convergence, which is about twice as many. *NMF*_*FCM*_ has the lowest convergence rate, with a mean number of iterations of 832. PG NMF shows the highest convergence rate for all initialization methods, with only *NMF*_*rRandom*_ requiring more than 100 iterations on average. Fig 2 illustrates the convergence behavior of the 4 single-level NMF methods when comparing initialization schemes for a particular glioblastoma patient. Initial errors are lowest for SPA and FCM. The main reduction of the residual error occurs within the first 100 iterations in all cases, except for Convex NMF with rRandom. For aHALS NMF, Convex NMF and PG NMF, all initialization methods converge to approximately the same residual error, whereas for GD NMF SPA and FCM converge to a higher final residual.

**Table 3:** Mean number of iterations to reach convergence for the different initialization methods on the UZ Ghent dataset. Convergence tolerance was set to 10^−5^ and the maximum number of iterations to 10000.

	#Iterations			
	SPA	NNDSVD	FCM	rRandom
**aHALS NMF**	179	181	140	190
**GD NMF**	218	175	832	418
**PG NMF**	84	80	85	109
**Convex NMF**	8923	8796	8819	8810

The software code used within the study has been made available in the manuscript’s Supporting Information files (see S1 File), along with one patient’s anonymized dataset. Interested researchers may run the code on this examplary dataset. Other patients included in the study did not consent to make their data publicly available.

## Discussion

### NMF performance

This study illustrates the behaviour of SPA as an initialization method for NMF in comparison to other common initialization methods for brain tumor segmentation. As noted in [1], there is no consensus on how to assess NMF performance when comparing different initialization methods. Many studies report the final residual error as a measure of performance [2, 3, 9], which has the advantage of being a generally applicable measure. Other studies use a validation which is specific to the problem at hand. Ortega-Martorell *et al*. calculated correlation coefficients as a quality measure of the obtained tissue sources for brain tumor characterization using single-voxel magnetic resonance spectroscopic data [13]. Wild *et al.*, besides looking at the residual error, assessed the quality of image sources for a facial image reconstruction problem based on visual inspection [6]. We assessed quality of the segmentation results using the Dice-scores, because a lower residual error does not warrant a better parts-based solution. For instance, one can observe in Fig 2 that higher residual errors remain for Convex NMF compared to aHALS and GD NMF. This is due to the additional constraint imposed by Convex NMF that the sources have to be a weighted sum of the input data. As can be seen in Table 1, Convex NMF reaches similar Dice-scores as the other single-level NMF methods. Therefore we decided to assess the quality of the NMF result using direct validation of the problem at hand, i.e. segmentation of the pathologic tumor regions. Mean Dice-scores did not differ by more than 5% for any NMF method using the different initialization strategies. In terms of the highest Dice-score per tissue class, SPA came out best on both datasets. SPA had the highest mean Dice-score in 12 out of 15 cases for the UZ Ghent dataset and in 10 out of 15 cases for the UZ Leuven dataset. These results suggest that SPA performs slightly better than the other initialization methods in terms of segmentation quality.

For most advanced initialization methods, similar performance has been reported compared to random initialization, although residual errors generally tend to be rather higher than lower. Wild *et al.* discussed how most advanced methods obtain a head start in terms of residual error compared to random initialization, but this advantage is mostly lost towards the end of the iteration process [6]. In some cases, random initialization will achieve a lower error towards the end of the convergence process, which is also illustrated in [6] and in Fig 2 for GD NMF. This is explained by the fact that advanced initialization might impose some restrictions on the factorization which favor other local minima, leading to more meaningful tissue segmentations. In Fig 2C, SPA and FCM show lower initial errors but higher final residual errors compared to rRandom and NNDSVD. The initial factor matrices obtained from SPA and FCM are more representative for the data at hand, and tend to converge to a nearby local optimum for GD NMF. As such, GD NMF is directed towards a factorization result which is meaningful in terms of tissue segmentation.

### Convergence and computational cost

Although a head start in terms of the initial error has been reported for most advanced initialization methods compared to random initialization, mixed results have been found in terms of the number of iterations to reach convergence [9]. As shown in Table 3, for aHALS NMF and Convex NMF we did not find considerable differences in convergence rate among the different initialization methods. For GD NMF, SPA and NNDSVD converge significantly faster than rRandom and FCM. For PG NMF, rRandom converges somewhat slower than the advanced initialization methods. Besides convergence speed, another important concern regarding computation time is the fact that random and FCM initialization are non-deterministic. Therefore, individual runs of these initialization strategies will not provide reproducible results, and some particular initializations might lead to a local optimum which is far from the global optimum. We decided to run NMF with repetitive random and FCM initialization, with a sufficient number of runs such that results were reproducible. This of course dramatically increased computation times for *NMF*_*rRandom*_ and *NMF*_*FCM*_.

Concerning computational complexity, SPA is a straightforward and computationally inexpensive method, requiring a total of 6*mnr* operations [16]. For NNDSVD, the main computational cost comes from calculating the truncated SVD of the input data matrix *X*. The SVD is commonly calculated by means of some iterative algorithm, with a computational complexity of *O(mnr)* operations per iteration [1]. This means that the leading constant, which depends on the number of iterations, will usually be much higher for NNDSVD compared to SPA. Similarly, FCM is an iterative procedure with a complexity of *O(mnr)* per iteration [9], such that the leading constant will be higher than for SPA. Mean computation times of the advanced initialization methods on the UZ Ghent dataset were 0.25s for NNDSVD and 4s for FCM. SPA took on average 0.02s to initialize *W*_0_. It must be noted, however, that both NNDSVD and FCM deliver *W*_0_ and *H*_0_, whereas SPA only delivers *W*_0_. In the current study, we used non-negative least squares fitting after SPA, taking on average 8s per patient to initialize *H*_0_. Computationally more efficient methods to initialize *H*_0_ are available. Random initialization is sometimes used for *H*_0_ in combination with a more advanced initialization of *W*_0_ [2], although this partially annihilates the advantages of using a fixed and input-specific initialization. Another possibility is to initialize *H*_0_ based on least-squares fitting without non-negativity constraints, then setting all negative values to zero or a small positive value. This actually comes down to one updating step of the basic block coordinate descent ALS algorithm. Standard least-squares will produce *H*_0_ with a higher initial error, but at a lower computational cost. Due to the relatively low cost of the initialization compared to the NMF computation itself, we did not explore more efficient strategies to initialize *H*_0_.

### SPA: Initialization or direct source extraction

The current study is not the first one to combine SPA with NMF. Several studies have applied SPA as an endmember extraction algorithm for NMF in hyperspectral unmixing [16, 31]. However, to the authors’ knowledge, SPA has so far not been considered as an initialization strategy. We hypothesized that there might be some benefit to assigning the SPA sources to *W*_0_ instead of directly using them as the final columns of *W*, because SPA will not always directly provide the most suitable sources for the NMF problem at hand. One of the main limitations of SPA is its sensitivity to outliers. As SPA looks for the outer vertices of a geometrical hull, outliers might be wrongly assigned as endmembers. Several methods have been proposed to either make SPA less sensitive to outliers [17] or to discard the outliers beforehand [32]. Another important caveat is that SPA assumes the NMF problem to be near-separable [16], which is equivalent to the spatial representation of the input data by a convex hull. The assumptions that are intrinsic to near-separable NMF, i.e. the pure-pixel assumption and the sum-to-one constraint on the abundances, will often not hold, especially in the case of highly mixed NMF problems. But when applying SPA as an initialization method, the NMF procedure might still correct the source estimates. For the multi-parametric MRI datasets considered in this study, the pure-pixel assumption holds for most of the MRI modalities due to their high spatial resolution. However, the poor resolution of the MR spectroscopic data leads to significant partial volume effects, such that the pure-pixel assumption does not hold for these data. We can further assume that the sum-to-one constraint approximately holds, as each voxel represents a fixed tissue volume. By comparing the Dice-scores of *NMF*_*SPA*_ to those obtained directly from SPA output matrices *W*_0_ and *H*_0_, it was shown that we can obtain better segmentation results when using SPA as an initialization method rather than as a direct source extraction tool. Mean SPA Dice-scores were lower than the Dice-scores obtained with any of the NMF methods and for all tissue classes. Statistical significance of the higher Dice-scores for *NMF*_*SPA*_ could be shown in about half of the cases for the UZ Ghent dataset and in most cases for the UZ Leuven dataset.

## Conclusion

This study has proposed SPA as an initialization method for NMF. Whereas SPA has previously been applied as a direct endmember extraction algorithm, using it as an initialization method might allow further enhancement of the sources during the NMF iterative procedure. This advantage has been shown on 2 multi-parametric MRI datasets for brain tumor segmentation. Compared to other initialization methods, SPA provides a realistic and reproducible initialization. Looking at the highest mean Dice-scores per NMF method and per tissue class, it was found that SPA performs slightly better than the other initialization methods in terms of segmentation quality. It is also competitive with the other initialization methods in terms of convergence rate. As the feasibility of using SPA initialization has been shown for multi-parametric MRI based tumor segmentation, we encourage the exploration of SPA in other NMF applications as well.

## Appendix

### Appendix 1: UZ Ghent dataset

Structural imaging included a 3-dimensional T1-weighted gradient-echo sequence (MPRAGE) with isotropic voxels (176 sagittal slices, field of view (FOV) read = 220mm, voxel size 0.9 × 0.9 × 0.9mm^3^, Repetition Time (TR) = 1550ms, Echo Time (TE) = 2.39ms, Inversion Time (TI) = 900ms, matrix size = 256 × 256, GRAPPA factor 2, flip angle = 9°) and a 3-dimensional T2-weighted inversion recovery sequence (FLAIR) with isotropic voxels (176 sagittal slices, FOV read = 250mm, voxel size 1 × 1 × 1mm^3^, TR = 6000ms, TE = 421ms, TI = 2100ms, matrix size = 256 × 238, GRAPPA factor 2). The MPRAGE sequence was repeated after administration of gadolinium contrast, namely following the acquisition of the Dynamic Susceptibility Contrast (DSC) perfusion-weighted imaging.

Perfusion-weighted imaging was performed by using a lipid-suppressed, T2*-weighted echo-planar imaging sequence with the following parameters: TR = 1000ms, TE = 29ms, FOV = 230 × 230mm^2^, slice thickness = 5.0mm, matrix size = 128 × 128, in-plane voxel size = 1.8 × 1.8mm^2^, 15 slices, distance factor = 30%, GRAPPA factor 2, flip angle = 90°. A series of 90 multi-section acquisitions was acquired at 1 second intervals. The first 10 acquisitions were performed before contrast agent injection to establish a pre-contrast baseline. At the tenth acquisition, a 0.1mmol/kg body weight bolus of gadobutrol (Gadovist, Bayer) was injected with a power injector (Spectris, Medrad Inc., Indianola, PA) at a rate of 4ml/s through a 18-gauge intravenous catheter, immediately followed by a 20 ml bolus of sodium chloride solution at 4ml/s. Relative Cerebral Blood Volume (CBV) maps were derived from the dynamic signal intensity curves in DSCoMAN (Duke University, Durham, NC) using the method proposed by Boxerman *et al*. [33].

Axial diffusion-weighted images were acquired using a fast single-shot gradient-echo echo-planar imaging sequence with diffusion gradient b-values of 0, 500 and 1000s/mm^2^ (voxel size 2.0 × 2.0 × 3.0mm^3^, TR = 5400ms, TE = 80ms, FOV read = 264mm, number of averages = 3). An affine coregistration was applied to account for eddy currents. Apparent Diffusion Coefficient (ADC) maps were derived from the 3 b-values using weighted linear least squares fitting [34]. The b0 images were also added to the dataset, serving as a T2-weighted reference.

A 3D proton magnetic resonance spectroscopic imaging (MRSI) protocol with long TE was included. In the two-slice MRSI examination, a volume of interest of 80 × 80mm^2^ including tumour, perilesional edema and normal brain tissue was positioned on reconstructions of the 3D FLAIR sequence. A Stimulated Echo Acquisition Mode (STEAM) pulse sequence was used for 3D spatial localization. The VOI was completely enclosed within the brain and 8 outer volume suppression slabs were placed outside the volume of interest. Magnetic resonance parameters were TR = 1700ms, TE = 135ms, flip angle = 90°, FOV = 160 × 160mm^2^, voxel size 10 × 10 × 15mm^3^, acquisition bandwidth 1200Hz, number of averages = 3. Weak water suppression was used. Automatic shimming with manual fine-tuning of the B_0_ magnetic field was used as well as iterative semi-automatic optimization of the transmitter voltage. The SPID software [35] was used to quantify the following metabolites with AQSES-MRSI [36]: lipid (Lip), lactate (Lac), N-acetyl aspartate (NAA), glutamine+glutamate (Glx), total creatine (Cre), total choline (Cho). Maximum phase FIR filtering was applied for residual water suppression [37]. One or two voxel bands at the outer edges of the spectroscopic grid were omitted from the analyses when showing severe chemical shift displacement effects or lipid contamination.

Twelve MRI imaging features were obtained from the raw acquired data after pre-processing: T1, T1+C, FLAIR, CBV, ADC, b0, Lip, Lac, NAA, Glx, Cre, Cho. In addition, 3 × 3 and 5 × 5 smoothing windows were applied to the 6 non-MRSI features, and these averaged features were also added to the feature set to improve robustness of the segmentation results [12]. A total of 24 MRI features was finally obtained for each voxel, making up the rows of the input matrix *X*.

### Appendix 2: UZ Leuven dataset

Structural MRI consisted of T2-weighted imaging, T1-weighted imaging with contrast enhancement and FLAIR. An axial spin echo T2-weighted MRI was acquired with the following parameters: TR/TE = 3000/80ms; slice/gap = 4/1mm; turbo factor = 10; FOV = 230 × 184*mm*^2^; acquisition matrix = 400 × 300. A T1-weighted 3D spoiled gradient echo MRI scan with contrast administration was performed with the following parameters: TR/TE/TI = 9.7/4.6/900ms; flip angle = 8°; turbo field echo factor = 180; acquisition voxel size = 0.98 × 0.98 × 1*mm*^3^; 118 contiguous partitions. An axial FLAIR MRI scan was acquired with the following parameters: TR/TE/TI = 11000/120/2800ms, slice/gap = 4/1 mm, FOV = 230 × 184*mm*^2^, acquisition matrix = 240 × 134.

Perfusion-weighted imaging was obtained using dynamic susceptibility-weighted contrast-enhanced MRI with a gradient-echo EPI sequence. A series of 60 multi-section scans was acquired at 1.35 second intervals, using the following parameters: TR/TE = 1350/30ms; section thickness/gap = 3/0mm; FOV = 200 × 200*mm*^2^; acquisition matrix = 112 × 109; EPI data were acquired during the first pass following a rapid injection of a 0.1mmol/kg body weight bolus of meglumine gadoterate (Dotarem, Guerbet, France) via a mechanical pump at a rate of 4ml/s, followed by a 20ml bolus of saline. Relative Cerebral Blood Volume (CBV) maps were derived from the dynamic signal intensity curves in DSCoMAN (Duke University, Durham, NC) using the method proposed by Boxerman *et al*. [33].

An extensive diffusion kurtosis imaging (DKI) protocol was included at UZ Leuven. A spin echo EPI diffusion-weighted sequence was used to acquire the DKI data. Implemented *b*-values were 0, 700, 1000, and 2800*s*/*mm*^2^, applied in respectively 1, 25, 40, and 75 uniformly distributed directions. The following parameters were used throughout the DKI acquisition sequence: TR/TE = 3200/90ms; gradient duration (*δ*) = 20ms; diffusion time interval (Δ) = 48.3ms; FOV = 240 × 240*mm*^2^; acquisition matrix = 96 × 96; number of signal averages = 1; section thickness/gap = 2.5/0mm; parallel imaging: SENSE with factor 2 in the antero-posterior direction. DKI parameters were estimated using a constrained weighted linear least squares (WLLS) model [34]. Mean diffusivity (MD), fractional anisotropy (FA) and mean kurtosis (MK) maps were computed from the DKI tensor as described in [38].

Proton MRSI consisted of a 2D short echo time sequence. Acquisition parameters were as follows: TR/TE = 2000/35ms; FOV = 160 × 160*mm*^2^; maximal region of interest = 80 × 80*mm*^2^; section thickness = 10mm; reconstruction voxel size = 5 × 5*mm*^2^; receiver bandwidth = 2000Hz; parallel imaging: SENSE. Automated pre-scanning optimized the shim in order to yield a peak width consistently under 20Hz full-width half-maximum. AQSES-MRSI was used to quantify the following metabolites: lipid (Lip), lactate (Lac), N-acetyl aspartate (NAA), glutamine+glutamate (Glx), total creatine (Cre), total choline (Cho), myo-inositol (mI) and glycine (Gly). Maximum phase FIR filtering was applied for residual water suppression [37]. One or two voxel bands at the outer edges of the spectroscopic grid were omitted from the analyses when showing severe chemical shift displacement effects or lipid contamination.

Fifteen MRI features were obtained from the raw acquired data after pre-processing: T2, T1+C, FLAIR, CBV, MD, FA, MK, Lip, Lac, NAA, Glx, Cre, Cho, mI and Gly. In addition, 3 × 3 and 5 × 5 smoothing windows were applied to the 7 non-MRSI parameters, and these averaged features were also added to the feature set to improve robustness of the segmentation results [12]. A total of 29 MRI features was finally obtained for each voxel.

## Supporting information

S1 File Software code. The software code used within the study has been made available in the file S1_File.zip, along with one patient’s anonymized dataset. Interested researchers may run the code on this examplary dataset. The code has been written in matlab. After unzipping the file, please consult the file README_Code.docx on how to run an NMF analysis and validate the segmentation result.
(ZIP)
