# Supplementary material for: The successive projection algorithm as an initialization method for brain tumor segmentation using non-negative matrix factorization
Source: PLoS One. 2017 Aug 28;12(8):e0180268. doi: 10.1371/journal.pone.0180268 (PMC5573288; doi:10.1371/journal.pone.0180268)
Supplement: S1 File — The software code used within the study has been made available in the file S1_File.zip, along with one patient’s anonymized dataset. Interested researchers may run the code on this examplary dataset. The code has been written in matlab. After unzipping the file, please consult the file README_Code.docx on how to run an NMF analysis and validate the segmentation result. (ZIP) [file pone.0180268.s001.zip › S1_file/Code/README_Code.docx]

**README file for Code**

The folder ‘Code’ contains the matlab code which has been used for performing NMF analyses with different initialization methods in the manuscript ‘The successive projection algorithm as an initialization method for brain tumor segmentation using non-negative matrix factorization’ (Sauwen N., et al.). The folder contains 4 subfolders:

*Initialization methods* code for the initialization methods

*NIFTI_tools* tools for loading nifti data into matlab

*NMF* code for importing the multi-parametric MRI input data and for running the NMF analyses

*Validation* code for validating the NMF segmentation result and for calculating the Dice scores

***Running NMF analyses***

The main function to call for NMF analyses is ‘NMF_initialized.m’ (in the subfolder ‘NMF’). Upon calling this function without input parameters, the user will first be asked to load the input data (demo data can be found in the folder ‘Demo data’). The initialization method to be used for initializing the factor matrices can be specified within the function ‘NMF_initialized.m’ (by default, it is set to SPA initialization). The parameter ‘nmfmethod’, which can also be set within the function, determines which NMF method will be used for the analyses. Code for running aHALS NMF is provided (‘HALSacc.m’) and is used by default. Code for running the other NMF methods is publicly available on the web, and can be downloaded from the following locations:

*Convex NMF* <https://sites.google.com/site/nmftool/>

*GD NMF* <http://www.tensorlab.net/>

*PG NMF* <https://www.csie.ntu.edu.tw/~cjlin/nmf/>

***Validation***

Code for visualizing the results and for calculating the Dice-scores is located in the subfolder ‘Validation’. The function ‘validate_NMF.m’ has to be called with the proper input parameters. Segmentation masks of the individual pathological tissue types have to be provided (in nifti format) when calling this function. For the demo data, these segmentation masks are available in the ‘Manual segmentation’ subfolder in ‘Demo data’.
