# Supplementary material for: The successive projection algorithm as an initialization method for brain tumor segmentation using non-negative matrix factorization
Source: PLoS One. 2017 Aug 28;12(8):e0180268. doi: 10.1371/journal.pone.0180268 (PMC5573288; doi:10.1371/journal.pone.0180268)
Supplement: S1 File — The software code used within the study has been made available in the file S1_File.zip, along with one patient’s anonymized dataset. Interested researchers may run the code on this examplary dataset. The code has been written in matlab. After unzipping the file, please consult the file README_Code.docx on how to run an NMF analysis and validate the segmentation result. (ZIP) [file pone.0180268.s001.zip › S1_file/Demo data/README_Data.docx]

**README file for demo data**

The folder ‘Demo data’ contains an examplary set of multi-parametric MRI data from a glioblastoma patient, acquired at the University Hospital of Ghent. Structural, perfusion-weighted and diffusion-weighted MRI features are provided in nifti (.nii) format and are all co-registered. The MR spectroscopic data (quantified metabolites) are spatially aligned with the nifti images and are provided in matlab (.mat) format. The MRSI volume of interest corresponds to the slice range ’45:54’ of the nifti files. This slice range will have to be specified upon loading the data, when running the code ‘NMF_initialized.m’ (see folder ‘Code’).

***Available files***

*rMPRAGEsagittaals003a1001.nii* T1-weighted images

*rMPRAGE+Csagittaalgds048a1001.nii* T1-weighted images with contrast enhancement

*rFLAIR3Dsags004a1001.nii* FLAIR images

*rCBV_DSC_denoised2.nii* rCBV images

*radc.nii* ADC images

*rDWIhighress005a1001_1.nii* b0 images

*Metab_maps_MRSI_3D.mat* MRSI data
